# Comparisons of Functional, Physical, and Mental Health Outcomes Among Young and Old Stroke Survivors

**DOI:** 10.3390/geriatrics11020024

**Published:** 2026-02-26

**Authors:** Molly M. Jacobs, Charles Ellis

**Affiliations:** 1 Health Services Research, Management and Policy, College of Public Health and Health Professions, University of Florida, Gainesville, FL 32610-0174, USA; mollyjacobs@phhp.ufl.edu; 2 Communication Equity and Outcomes Laboratory, Department of Speech, Language and Hearing Sciences, College of Public Health and Health Professions, University of Florida, Gainesville, FL 32610-0174, USA

**Keywords:** stroke, functional disability, mental health, employment status, young adults, BRFSS, Poisson regression, logistic regression

## Abstract

**Objective:** The objective of this study was to examine how functional, mental, and physical health outcomes differ between younger (<age 50) and older (≥age 50) stroke survivors. **Methods:** Data from adult stroke survivors examined health-related outcomes (physical and mental health) over the past 30 days. Logistic regression models were used for binary functional outcomes, and Poisson regression models were used to estimate count outcomes for poor mental and physical health days. **Results:** Compared with older adults, younger stroke survivors were more likely to report difficulty concentrating or remembering (41.1% vs. 23.2%, *p* < 0.0001) and difficulty doing errands alone (27.11% vs. 23.67%, *p* = 0.00), but less likely to report difficulty walking or climbing stairs (34.3% vs. 47.6%, *p* < 0.0001). Additionally, younger adults with stroke reported significantly more poor mental health days (10.81 vs. 5.76, *p* < 0.0001) than older adults. In adjusted models, being out of work or out of the labor force was consistently associated with greater odds of functional limitations (e.g., OR for activity difficulty = 2.07, 95% CI: 1.56–2.75) and higher counts of poor mental and physical health days. Younger stroke survivors who were out of the labor force had significantly greater odds of difficulty concentrating (OR = 2.02, 95% CI: 1.17–3.48) and increased days of poor mental (IRR = 1.27, 95% CI: 1.19–1.70) and physical health (IRR = 1.26, 95% CI: 1.19–1.53). **Conclusions:** These findings highlight the intersection of age and employment on stroke outcomes. Younger stroke survivors face unique and disproportionate challenges in functional and mental health.

## 1. Background

Stroke is the third leading cause of long-term disability worldwide [1]. Although most common among older adults, stroke among young adults (age 49 and below) has increased worldwide, particularly in low- and middle-income countries [2]. The underlying cause of stroke and associated risk factors among young adults varies dramatically from older and more traditional age individuals [3]. Young adults are more likely to have strokes that are attributed to illicit drug use, pregnancy, arterial dissections, and patent foramen ovale (PFO) [3]. Because the mechanisms underlying stroke differ in young adults compared with older adults, they also exhibit different clinical presentations, thereby making management more challenging [4].

Young adults with stroke can experience similar levels of physical disability, cognitive disability, and depression as older adults, which translates into a loss of productivity to society at a very young age [5]. Many report challenges with mobility, self-care, and limitations in their ability to engage in leisure activities and return to work [6]. A large percentage will not return to pre-stroke employment, mainly due to physical disability [7]. Consequently, understanding the prognosis of young adults with stroke has become of great concern given their likelihood of a long life expectancy [8]. Additionally, an urgent need has emerged to establish secondary stroke prevention strategies for young adults with stroke [9,10].

Because the underlying causes and outcomes of stroke in young adults differ from those of older adults, their management strategies and needs also vary. Young adults with stroke report unmet needs associated with a lack of person-centered care appropriate for their individual needs and life stage [11]. Some report feeling that healthcare systems are fragmented and frequently overlook the unique needs of young stroke survivors [12]. Regarding rehabilitation, a recent review noted that rehabilitation units are not age-adapted for young stroke survivors [13]. Additionally, young adult stroke survivors report a greater need for face-to-face communication with healthcare providers, staff peer support, clear information about their recovery, and focused support for non-physical impairments [14]. Finally, young stroke survivors note experiencing challenges and a lack of appropriate services, such as longitudinal medical follow-up to address their unique needs and resources to assist them in reintegrating into their communities [15].

Some of the challenges in addressing the unique needs of young stroke survivors may lie in a lack of clear understanding of how their needs differ from those of older and more traditional age individuals experiencing stroke. Consequently, young adult stroke survivors enter systems designed for older adults that may not address their unique needs. To date, there is evidence in the literature describing differences between younger and older adults in incidence [9,16,17], risk factors [17,18], presenting symptoms [19], mortality [17,18], stroke reoccurrence [20], outcomes at hospital discharge [18], and economic burden [21]. However, few if any reports have emerged comparing patient perceptions of their health and functionality post-stroke, such as health status, life satisfaction, and functional performance in walking/climbing, dressing/bathing, or running errands. Similarly, little is known about their ability to meet their emotional support needs, secure adequate transportation, return to work, and subsequently paying their bills. To explore these issues, we utilized data collected from the Centers for Disease Control and Prevention’s (CDC) Behavioral Risk Factor Surveillance System Survey (BRFSS) from U.S. adults who reported a history of stroke.

## 2. Methods

### 2.1. Data

Data for this study were drawn from the 2023 BRFSS, a nationally representative, cross-sectional survey administered annually by the CDC in collaboration with U.S. states and territories. The BRFSS is the largest continuously conducted health survey system in the world, collecting data on health-related risk behaviors, chronic health conditions, and use of preventive services among non-institutionalized adults aged 18 years and older in the U.S.. The 2023 BRFSS employed a multistage, stratified sampling design that utilized random digit dialing techniques for both landline and cellular phones. The survey was administered in all 50 states, the District of Columbia, and participating U.S. territories. Complex survey design variables—including strata, primary sampling unit (PSU), and individual-level sampling weights—were provided by the CDC and used to ensure nationally representative estimates and to account for nonresponse bias, oversampling, and disproportionate probabilities of selection.

The 2023 BRFSS core questionnaire included modules on sociodemographic characteristics, health status, healthcare access, and chronic conditions, including a self-reported history of stroke. Additional questions assessed functional limitations, mental health (e.g., number of poor mental health days in the past 30 days), and physical health (e.g., number of physically unhealthy days). These items are standardized across states, allowing for consistent comparisons across geographic and demographic groups. All BRFSS data are publicly available and de-identified. Therefore, this study was deemed exempt from Institutional Review Board (IRB) approval under federal guidelines for the secondary analysis of public datasets.

### 2.2. Sample

The sample was limited to (1) those individuals who indicated that they had been told by a doctor, nurse, or other healthcare professional that they had a stroke (18,350) and (2) those who replied to questionnaire items related to functional difficulties (413,923). The resulting analytic sample consisted of 17,756 respondents.

### 2.3. Functional Abilities

Four functional health outcomes were examined to assess the extent of disability among stroke survivors. These outcomes were based on self-reported difficulty with core activities of daily living (ADLs) and instrumental activities of daily living (IADLs), as measured in the BRFSS. Specifically, respondents were asked whether they experienced: (1) difficulty concentrating, remembering, or making decisions due to a physical, mental, or emotional condition; (2) difficulty walking or climbing stairs; (3) difficulty dressing or bathing; and (4) difficulty doing errands alone, such as visiting a doctor’s office or shopping. Each outcome was treated as a binary variable indicating the presence or absence of the functional limitation. These items provide a multidimensional view of cognitive, mobility, and self-care challenges, offering insight into the everyday functional burdens experienced by stroke survivors across different age groups.

### 2.4. Mental/Physical/Poor Health Days

Mental and physical health outcomes were measured using standardized items from the 2023 BRFSS that assess the frequency of health-related distress and activity limitation. Respondents were asked: (1) “Now thinking about your mental health, which includes stress, depression, and problems with emotions, for how many days during the past 30 days was your mental health not good?”; (2) “Now thinking about your physical health, which includes physical illness and injury, for how many days during the past 30 days was your physical health not good?”; and (3) “During the past 30 days, for about how many days did poor physical or mental health keep you from doing your usual activities, such as self-care, work, or recreation?” Responses to each item ranged from 0 to 30 days, with higher values indicating a greater number of days with poor health or activity limitation. These variables were analyzed as continuous outcomes to reflect the burden of impaired well-being and functioning. Together, they provide a subjective yet meaningful measure of recent health-related quality of life, capturing distinct but interrelated dimensions of mental distress, physical illness, and the extent to which these issues interfere with daily life.

### 2.5. Demographic Characteristics

Demographic characteristics were collected using standardized survey items. Age was reported by respondents in years and top-coded at 80 in the publicly available dataset. Stroke survivors below age 50 were considered “young.” Sex was primarily coded by interviewers as either male or female based on vocal cues, including the respondent’s name and tone of voice. If the interviewer was unable to determine the respondent’s sex with confidence, the respondent was asked directly, “I’m required to ask, are you male or female?”

Race and ethnicity were collected using a two-step process. Respondents were first asked whether they identified as Hispanic, Latino/a, or of Spanish origin. They were then asked to select one or more racial categories from the following: White, Black or African American, American Indian or Alaska Native, Asian, Native Hawaiian or Other Pacific Islander, or Other. In the final public use dataset, responses are recoded into a single race/ethnicity variable using standardized CDC protocols, with a priority given to Hispanic ethnicity and single-race categories for consistency in reporting. The national sample consisted of only 2.83% Non-Hispanic Asian, 1.60% American Indian/Alaskan Native, Non-Hispanic, and 3.70% Non-Hispanic Other race. These groups had even smaller representation among stroke survivors; thus, they were combined into a single cohort.

Marital status was reported as married, divorced, widowed, separated, never married, or a member of an unmarried couple; however, these categories were collapsed into two main categories: married and not married. Educational attainment was determined by asking the respondent about the highest grade or year of school they had completed. Response options included less than a high school education, a high school graduate or GED, some college or technical school, and a college graduate. These were coded as less than high school, high school or some college, and college or above.

All respondents indicated whether they were insured at the time of the interview and selected the category appropriate to their annual pre-tax household income. BRFSS categories included less than $10,000, $10,000 to less than $15,000, $15,000 to less than $20,000, $20,000 to less than $25,000, $25,000 to less than $35,000, $35,000 to less than $50,000, $50,000 to less than $75,000, and $75,000 or more. In this study, household income was dichotomized into a binary variable (<$50,000 vs. $50,000 or more). The $50,000 threshold approximates 200% of the federal poverty level for a family of four, a commonly used benchmark in determining eligibility for public assistance programs and the Affordable Care Act [22]. Additionally, the U.S. Census Bureau frequently uses $50,000 as a midpoint in income distribution tables, marking the lower boundary of middle-income households [23]. Dichotomizing income at this threshold simplifies interpretation, improves model parsimony, and aligns with prior public health literature that uses similar cutoffs to assess financial vulnerability and its association with health outcomes.

Respondents reported their employment status as employed for wages, self-employed, out of work for 1 year or more, out of work for less than 1 year, a homemaker, a student, or retired. These categories were collapsed into employed (including working for wages and self-employed), unemployed (including out of work for ≥1 year and out of work for <1 year), and out of the labor force (including homemakers, students, and retirees).

### 2.6. Statistical Analysis

Statistical analyses were conducted to examine differences in health outcomes between younger and older stroke survivors. Logistic regression models were used to estimate the association between age at stroke onset and each of the four binary functional outcomes, while Poisson regression models were employed to estimate the number of poor mental and physical health days reported in the past 30 days. The primary independent variable of interest was a binary indicator for age under 50 years, representing “young stroke” survivors. All models were adjusted for key demographic covariates, including sex, marital status, race/ethnicity, insurance coverage, educational attainment, and employment status (employed, unemployed, or out of the labor force), with employment serving as the reference category.

Since age and employment may interact to influence health outcomes, as younger stroke survivors may face unique challenges in returning to work or maintaining employment compared with their older counterparts, an interaction term between age less than 50 and employment status was included in each model. The interaction term enabled the identification of differential effects of employment status between young and older stroke survivors, capturing a potentially important dimension of post-stroke recovery and functional limitations. All analyses accounted for the complex survey design of the BRFSS, including sampling weights, strata, and primary sampling units, to ensure population-level inference.

## 3. Results

### 3.1. Descriptive Characteristics

Table 1 presents descriptive characteristics of the analytic sample of stroke survivors (17,756), stratified by age at stroke onset. Young stroke survivors (age < 50) represented 9.35% of the sample (1661), while the majority (90.65%) were age 50 or older (16,095). Notable demographic differences existed between the two groups. Compared with older stroke survivors, those under age 50 were more likely to be female (55.93% vs. 52.32%, *p* = 0.01), not married (64.54% vs. 56.78%, *p* < 0.0001), and uninsured (11.95% vs. 1.91%, *p* < 0.0001). Younger stroke survivors were also more racially and ethnically diverse, with higher proportions identifying as Black (11.62% vs. 10.13%), Hispanic (16.98% vs. 4.83%), or Other race (15.17% vs. 7.37%) compared with older survivors (*p* < 0.0001). Educational attainment differed significantly by age group, with younger adults more likely to have less than a high school education (*p* < 0.0001). No significant differences were observed by income category.

### 3.2. Functional Abilities

Younger stroke survivors were more likely to report difficulty concentrating, remembering, or making decisions (41.12% vs. 23.22%, *p* < 0.0001) and difficulty doing errands alone (27.11% vs. 23.67%, *p* = 0.00). Still, they were less likely to report difficulty walking or climbing stairs (34.32% vs. 47.64%, *p* < 0.0001). No significant difference was found in difficulty dressing or bathing between the two groups (*p* = 0.35). See Table 1.

### 3.3. Poor Physical/Poor Mental/Poor Total Health Days

On average, young stroke survivors reported significantly more poor mental health days in the past 30 days compared with their older counterparts (mean = 10.81 vs. 5.76, *p* < 0.0001). However, no statistically significant differences were observed in reported poor physical health days (*p* = 0.00) or total poor health days (*p* = 0.31). See Table 1.

### 3.4. Multivariable Logistic Regression Models—Associations with Functional Abilities

Table 2 presents the results of multivariable logistic regression models examining associations of four types of functional abilities among stroke survivors. Employment status emerged as one of the most robust and consistent associations of post-stroke functional limitations. Compared with individuals who were employed, those who were out of work had significantly higher odds of difficulty concentrating, remembering, or making decisions (OR = 1.77, 95% CI: 1.07–2.92), difficulty walking or climbing stairs (OR = 2.04, 95% CI: 1.31–3.17), difficulty dressing or bathing (OR = 2.14, 95% CI: 1.16–3.95), and difficulty doing errands alone (OR = 2.07, 95% CI: 1.19–3.60). Similarly, those who were out of the labor force—which includes retirees, students, and people unable to work—had even greater odds of these limitations across all domains, with the strongest association observed for difficulty doing errands alone (OR = 3.55, 95% CI: 2.36–5.32).

Importantly, interaction terms revealed that the effect of employment status varied significantly by age. Among younger stroke survivors (<age 50), being out of the labor force was associated with significantly elevated odds of difficulty concentrating or remembering (OR = 2.02, 95% CI: 1.17–3.48), difficulty walking or climbing stairs (OR = 1.97, 95% CI: 1.14–3.41), and difficulty doing errands alone (OR = 2.15, 95% CI: 1.11–4.15). These interactions suggest a compounding disadvantage for young stroke survivors who are not engaged in the workforce.

In contrast, younger age alone (<50) was associated with lower odds of difficulty walking or climbing stairs (OR = 0.49, 95% CI: 0.32–0.74). Still, no significant associations were observed between young age and the other three functional outcomes in the main effects. This suggests that the intersection of age and employment status, rather than age alone, plays a significant role in shaping functional limitations after stroke. These findings underscore the importance of incorporating labor force engagement into models of stroke recovery and highlight the need for targeted interventions that support workforce reintegration for younger stroke survivors.

### 3.5. Poisson Regression Models

Associations with Mental, Physical, and Poor Health Days: Table 3 presents results from Poisson regression models examining the number of days in the past 30 days that stroke survivors reported poor mental health, poor physical health, and poor health days or activity limitations due to health. Employment status was a significant and consistent association across all three outcomes. Compared with employed individuals, those out of work reported significantly more days of activity limitation (IRR = 2.07, 95% CI: 1.56–2.75), poor physical health (IRR = 1.67, 95% CI: 1.34–2.08), and marginally more poor mental health days (IRR = 1.29, *p* = 0.09). Those out of the labor force—including retirees, disabled individuals, and others not seeking work—also had higher counts of activity limitation days (IRR = 1.72, 95% CI: 1.35–2.19) and poor physical health days (IRR = 1.46, 95% CI: 1.24–1.71) and showed a modest but significant interaction effect with younger age for poor mental and physical health.

The interaction between younger age (<50) and being out of the labor force was statistically significant for all three outcomes: activity limitation (IRR = 1.32, 95% CI: 1.23–1.86), poor mental health days (IRR = 1.27, 95% CI: 1.19–1.70), and poor physical health days (IRR = 1.26, 95% CI: 1.19–1.53). These findings suggest that being detached from the labor force is particularly detrimental for younger stroke survivors, exacerbating both mental and physical health burdens.

Other Demographic Factors Associated with Reduced Functional Abilities and Poor Mental, Physical, and Total Poor Health Days: Low income was linked with more days of activity limitation (IRR = 1.23, 95% CI: 1.06–1.41), poor mental health (IRR = 1.25, 95% CI: 1.08–1.45), and poor physical health (IRR = 1.26, 95% CI: 1.12–1.41). Higher educational attainment was protective: high school graduates had significantly fewer days of activity limitation (IRR = 0.78, 95% CI: 0.68–0.89), poor mental health (IRR = 0.81, 95% CI: 0.70–0.94), and poor physical health (IRR = 0.87, 95% CI: 0.78–0.97) compared with those with less than a high school education. College graduates showed even stronger protective effects across all outcomes: activity limitation (IRR = 0.76, 95% CI: 0.64–0.89), mental health (IRR = 0.63, 95% CI: 0.53–0.75), and physical health (IRR = 0.81, 95% CI: 0.71–0.93).

Marital status was associated with better mental health outcomes, with married respondents reporting fewer poor mental health days (IRR = 0.81, 95% CI: 0.72–0.92). However, this effect was not statistically significant for physical health or activity limitation. Younger age (<50) was associated with more poor mental health days (IRR = 1.37, 95% CI: 1.07–1.74), but not with significant differences in physical health or activity limitation.

Taken together, these findings underscore the critical role of employment status, especially being out of the labor force, as a key driver of poor health days, particularly among younger stroke survivors. The interaction between age and labor force status suggests that younger individuals not working may experience an amplified health burden, highlighting the importance of post-stroke vocational rehabilitation and social support interventions targeted to this high-risk group.

## 4. Discussion

Studies of stroke have shown an increase in stroke rates among young adults in many countries, even though rates among older adults have declined [2]. Some aspects of the stroke experience and associated outcomes among young adults are like older adults. At the same time, other areas differ significantly due to distinct and, in some cases, more prominent unique challenges [11,12,15,24]. In this study of over 17,000 stroke survivors, of whom 1661 experienced their stroke before age 50, significant differences were observed when they were compared with older adults with stroke. Four unique findings emerged from this work. First, young adults with stroke were more likely to report difficulty concentrating or remembering and doing errands alone, but less likely to report difficulty walking or climbing stairs when compared with older adults. This finding suggests that cognitive functional abilities are more difficult than physical functional abilities when compared with those of older adults. Second, younger adults with stroke reported significantly more poor mental health days than older adults. Third, being unemployed or out of the workforce was associated with worse functional and mental health outcomes. More specifically, young stroke survivors who were not working had greater odds of reduced functional abilities (e.g., activity difficulty) and a higher number of reported days of poor mental and physical health. Additionally, lack of employment magnified their adverse outcomes as young stroke survivors who were out of the labor force reported greater odds of difficulty concentrating, and the number of days of poor mental and physical health was higher. Fourth, higher education was associated with more positive outcomes, whereas low income was associated with worse outcomes and health status. Given the complexity and significance of these findings, we will first explore each finding and then examine potential synergistic and additive impacts of these outcomes in young stroke survivors.

### 4.1. Differences in Reported Reductions in Functional Abilities

Young stroke survivors in this study were more likely to report difficulty concentrating or remembering and running errands independently, yet they were less likely to report difficulty walking or climbing stairs. These findings are critically important as they highlight differences in outcomes of young adults when compared with older adults. The issue of running errands is interesting as the completion of errands requires functional executive skills beyond the simple recall of tasks to be completed. A recent review noted that disruptions in executive skills are common after stroke and can result in significant restrictions in pre-stroke community participation even among individuals with mild stroke [25].

The findings of this study align with previous work suggesting that not only are the outcomes of young stroke survivors different from older adults, but in some cases, they are distinct and unique [12,15]. According to Huang and colleagues, many young adults with stroke experience unique challenges, some of which can be attributed to gaps in rehabilitative services designed to address the specific deficits of older adults [15]. Thompson et al. noted that rehabilitative care remains focused on the needs of older adults and oftentimes fails to address deficits such as cognition and communication in young stroke survivors [12]. Consequently, their specific needs are not adequately addressed, leaving some individuals feeling alienated from re-entering society [12].

Notably, differences in rehabilitative care needs between young and older adults are not straightforward. Symptom variability can differ between stroke onset in younger adults and older adults, which can be attributed to differences in the underlying causes of stroke and the stroke subtypes in young and older adults [9]. Sič and colleagues note that it is critically important to understand that stroke in young adults is not simply a milder version of the stroke disease condition in older adults but a dramatically different clinical entity [10]. Finally, understanding age-related differences in functional ability is critically important when considering that functional impairment is the primary factor that precludes return to independence, particularly in young stroke survivors [26].

### 4.2. More Reported Poor Mental Health Days

Like differences in functional ability, young stroke survivors were more likely to report poor mental health days. It is known that individuals with disabilities are more likely to experience mental distress and other mental health issues than individuals without disabilities [27]. Stroke survivors of all ages can experience emotional changes after a stroke. Because physical disability after stroke is the most overt outcome, mental health issues are oftentimes underrecognized [28]. Mental health issues such as emotional distress and mood disturbances such as depression and anxiety occur in roughly one-third of all stroke survivors [29]. Given these estimates, it is not surprising that stroke survivors would report poor mental health days each month. However, the differences between young and old stroke survivors are striking (10.81 days vs. 5.76 days). One prior study examining the overall health of stroke survivors 18 to 64 years old found that 21% of the sample reported ≥14 unhealthy mental days [30]. By comparison, <10% of the general population of adults reported that their mental health was not good for ≥14 days during the past 30 days [31].

The underlying neurophysiological mechanisms that support stroke recovery are complex and are believed to result in behavioral changes that can negatively impact mental health [32]. However, the specific mechanisms are poorly understood, and consequently, the potential impact of stroke on reported mental health days is not clear. Less is known about mental health days among young adults with and without stroke. One study using BRFSS data explored poor mental health days among 593,616 young adults, aged 18 to 49 years, and found that 29.2% reported 1 to 13 days of poor mental health in the past 30 days and 15.1% reported ≥14 days of poor mental health in the past 30 days [33]. However, to date, the findings reported here have few if any comparative studies to explore further the number of days among all stroke survivors and how those numbers potentially differ between young and old adults.

### 4.3. Out of Work/Labor Force Associated with Worse Functional Abilities and Poorer Mental/Physical Health

It is not a surprise that young stroke survivors who were not employed had worse functional skills and reported more poor mental and physical health days. Return to work after stroke remains a challenge for all stroke survivors. However, most prior research has been conducted in older adults, and thus, little is known about young adults [7]. The requirements for returning to work after stroke potentially differ between old and young adults, given their differences in life stage, employment stage, and skill level [7]. For the same reason, returning to work may be of greater central importance for young adults [7]. According to Maaijwee et al., young adults with stroke are nine times more likely to be unemployed than individuals their same age in the general population [8]. Therefore, young stroke survivors face greater and longer-term economic burdens associated with being unemployed and face increased life expectancy with stroke than older stroke survivors [8,10]. The economic burden of stroke in young adults extends beyond the young stroke survivor but also to their families and society due to their lack of workforce participation during their productive years [34].

In addition to the economic burden, returning to work is associated with greater life satisfaction after stroke [35]. While employment status emerged as a strong and consistent correlate of functional and mental health outcomes, the cross-sectional nature of the data precludes establishing directionality. The relationship between employment, functional limitation, and mental health is likely bidirectional and mutually reinforcing. Functional impairments may limit an individual’s ability to maintain employment, while job loss or labor force detachment may contribute to worsening psychological distress, financial strain, and reduced access to employer-sponsored resources that support recovery. In younger stroke survivors in particular, identity formation, financial independence, and social integration are often closely tied to employment status, potentially amplifying the psychological consequences of workforce disengagement. Conversely, poor mental health may impede return-to-work efforts and exacerbate perceived functional difficulty. These interdependent pathways suggest that employment should not be viewed solely as a predictor of health outcomes, but rather as part of a dynamic recovery process requiring integrated vocational, psychological, and rehabilitative interventions. Therefore, return to work along with overall community reintegration and establishment of overall independence must be a focus of rehabilitative efforts for young adults with stroke [6].

Education and income are protective factors for stroke outcomes. Individuals from low educational backgrounds have a higher risk of initial and recurrent stroke [36]. Similarly, higher educational attainment appears to have a protective effect on stroke risk [37,38]. Therefore, the finding that education has a protective effect on stroke outcomes in young adults is not surprising. Education is highly correlated with income and socioeconomic status (SES), and therefore, the finding of worse functional outcomes among young stroke survivors at the lowest income levels is also supported by the current stroke literature. Studies show that lower income and SES are associated with increased stroke risk [39]. Ngueyn et al. [39] found that individuals from low-income backgrounds had the highest prevalence of stroke, regardless of whether they were young adults or middle-aged. Additionally, low income is associated with greater mortality and post-stroke disability [40]. Therefore, greater attention and potentially targeted intervention must be given to young adults with stroke from low educational and income backgrounds to improve their outcomes.

### 4.4. Limitations

There are several limitations to this study. Primarily, the BRFSS is a cross-sectional, self-report survey, and therefore is subject to recall and social desirability bias, which may influence the events respondents recall or report at the time of response. Response rates for the BRFSS can vary between states. Because the survey administration differs slightly between landline and cellphone interviews, some unobserved response bias may be introduced into the sample. Additionally, some population groups are oversampled, resulting in higher estimates than expected. The samples included in BRFSS data represent unweighted frequencies, and when sample weights are applied, the sample proportions are adjusted to best match the population of interest. Thus, it is essential to consider that these are the raw frequency values that reflect basic survey response patterns, and some degree of caution should be considered in interpreting the raw values. Furthermore, logistic regression models comprised the primary form of data analysis. While logistic regression can be easily interpreted and provides an accurate measure of both appropriateness and direction of association, it relies on the assumption of linearity between the dependent and independent variables to satisfy the log odds assumption. Logistic regression also requires no or minimal multicollinearity between independent variables—a test that is confounded by the complex statistical controls required to analyze BRFSS data appropriately.

Additionally, several groups were collapsed into the ‘Other’ racial/ethnic category to avoid removing these individuals from the sample. However, this resulted in a highly heterogeneous group, making it difficult to generalize to the larger population. Finally, BRFSS does not collect the age at the time of stroke. Therefore, the age at the time of interview was used to create the young and old stroke groups. We acknowledge that respondents included in the older stroke category could have had a stroke below age 50 and not been interviewed by BRFSS until later in life. Furthermore, age and income were operationalized as dichotomous variables to enhance interpretability and model parsimony. Young stroke was defined as age < 50 years, consistent with prior literature and epidemiologic conventions distinguishing early-onset stroke from more traditional, later-onset presentations. However, alternative cut points (e.g., <45 years or <55 years) may yield modest differences in effect magnitude, particularly given the gradient of stroke risk and functional decline across midlife. Similarly, household income was dichotomized at $50,000 to approximate economic vulnerability; nonetheless, alternative thresholds or modeling income as an ordinal or continuous measure could capture additional heterogeneity in socioeconomic gradients. While dichotomization facilitates interpretation and policy relevance, future research should examine whether associations between age, income, and post-stroke outcomes demonstrate nonlinear or threshold effects across more granular categories. Finally, detailed clinical information is not obtained in the BRFSS survey; therefore, stroke severity and stroke subtype cannot be carefully considered in relationship to the outcomes measured.

## 5. Conclusions

Stroke among young adults is relatively uncommon, yet prevalence rates have been increasing recently. Traditionally, studies of stroke have emphasized older, more traditional age individuals experiencing a stroke. Stroke among young adults mirrors some aspects of stroke in older adults; however, their younger age and differing life status translate into unique clinical management issues that require alternative approaches. Future studies should compare stroke outcomes of young adults with older adults while also attempting to address the uniqueness of young adults who can experience this disabling condition. Those studies should consider clinical variables such as stroke type and associated cerebrovascular disease contributions to outcomes [40]. Future studies should also consider targeted interventions to address the unique needs of both young and older stroke survivors. Targeted interventions should consider issues beyond rehabilitation but also the unique vocational and community reintegration needs of young and old stroke survivors in addition to constantly evolving technologies designed to improve stroke outcomes [18,41,42]. In addition to age, differential patterns between men and women should be considered particularly given reports to sex differences in stroke-related risk factors and stroke outcomes [43].

## Figures and Tables

**Table 1:** Sample characteristics of stroke survivors.

	Full Sample (*N* = 17,756)		Young Stroke, Age < 50 (*N* = 1661, 9.35%)		Old Stroke, Age ≥ 50 (*N* = 16,095, 90.65%)			
	*N*	PCT	*N*	PCT	*N*	PCT	χ^2^-Stat	*p*-Value
Male	8406	47.34	732	44.07	7674	47.68	7.87	0.01
Female	9350	52.66	929	55.93	8421	52.32		
Less than high school	7061	39.77	228	13.73	1503	9.34	17.59	<0.0001
High school/Some college	10,695	60.23	1009	60.75	9686	60.18		
College or above	5330	30.02	424	25.53	4906	30.48		
Not married	10,211	57.51	1072	64.54	9139	56.78	37.08	<0.0001
Married	7545	42.49	589	35.46	6956	43.22		
White	13,435	75.66	934	56.23	12,501	77.67	580.33	<0.0001
Black	1823	10.27	193	11.62	1630	10.13		
Other	1439	8.1	252	15.17	1187	7.37		
Hispanic	1059	5.96	282	16.98	777	4.83		
Not insured	484	2.85	190	11.95	294	1.91	523.41	<0.0001
Insured	16,472	97.15	1400	88.05	15,072	98.09		
Less than $50,000	8808	62.1	861	61.19	7947	62.2	0.54	0.46
$50,000 or more	5376	37.9	546	38.81	4830	37.8		
Difficulty concentrating or remembering	4421	24.9	683	41.12	3738	23.22	257.85	<0.0001
Difficulty doing errands alone	4252	23.99	449	27.11	3803	23.67	9.76	0.00
Difficulty dressing or bathing	2617	14.74	232	13.97	2385	14.82	0.87	0.35
Difficulty walking or climbing stairs	8238	46.4	570	34.32	7668	47.64	107.50	<0.0001
	Mean	SD	Mean	SD	Mean	SD	F-Stat	*p*-Value
Physical health	10.11	12.24	9.97	11.60	10.13	12.30	1.12	0.00
Mental health	6.23	10.26	10.81	11.85	5.76	9.96	1.42	<0.0001
Poor health	10.04	11.91	10.35	11.63	10.00	11.94	−1.01	0.31

**Table 2:** Functional difficulties among stroke survivors.

Difficulty concentrating, remembering, or making decisions							
		Std Err	t-Stat	*p* > |t|	Odds Ratio	95% Confidence Interval	
Female	**0.20**	0.08	2.39	0.02	1.23	1.04	1.45
High school graduate	**−0.62**	0.12	−5.22	0.00	0.54	0.43	0.68
College graduate	**−1.03**	0.15	−7.01	0.00	0.36	0.27	0.48
Married	−0.05	0.09	−0.50	0.62	0.95	0.79	1.15
Age < 50	0.28	0.22	1.28	0.20	1.33	0.86	2.05
Black	−0.09	0.13	−0.71	0.48	0.91	0.71	1.17
Hispanic	0.12	0.16	0.76	0.45	1.13	0.83	1.54
Other race	**0.51**	0.16	3.15	0.00	1.66	1.21	2.28
No insurance	0.33	0.22	1.49	0.14	1.40	0.90	2.16
Low income	**0.54**	0.11	4.76	0.00	1.72	1.38	2.15
Out of work	**0.57**	0.26	2.23	0.03	1.77	1.07	2.92
Out of the labor force	0.21	0.16	1.31	0.19	1.24	0.90	1.69
Age < 50 out of work	−0.25	0.43	−0.59	0.56	0.78	0.34	1.79
Age < 50 out of the labor force	**0.70**	0.28	2.53	0.01	2.02	1.17	3.48
Intercept	**−1.23**	0.21	−5.73	0.00	0.29	0.19	0.45
Difficulty walking or climbing stairs							
		Std Err	t-Stat	*p* > |t|	Odds Ratio	95% Confidence Interval	
Female	**0.22**	0.08	2.87	0.00	1.24	1.07	1.44
High school graduate	**−0.45**	0.11	−3.89	0.00	0.64	0.51	0.80
College graduate	**−0.81**	0.13	−6.12	0.00	0.44	0.34	0.58
Married	**−0.24**	0.08	−2.90	0.00	0.79	0.67	0.93
Age < 50	**−0.72**	0.21	−3.40	0.00	0.49	0.32	0.74
Black	0.09	0.12	0.76	0.45	1.09	0.87	1.38
Hispanic	0.13	0.15	0.88	0.38	1.14	0.85	1.53
Other race	**0.58**	0.17	3.37	0.00	1.79	1.27	2.50
No insurance	−0.02	0.21	−0.10	0.92	0.98	0.65	1.47
Low income	**0.49**	0.10	4.92	0.00	1.63	1.34	1.98
Out of work	**0.71**	0.22	3.18	0.00	2.04	1.31	3.17
Out of the labor force	**1.06**	0.12	8.54	0.00	2.89	2.27	3.69
Age < 50 out of work	−0.14	0.60	−0.23	0.82	0.87	0.27	2.84
Age < 50 out of the labor force	**0.68**	0.28	2.42	0.02	1.97	1.14	3.41
Intercept	**−0.88**	0.18	−4.79	0.00	0.41	0.29	0.59
Difficulty dressing or bathing							
		Std Err	t-Stat	*p* > |t|	Odds Ratio	95% Confidence Interval	
Female	−0.10	0.10	−0.93	0.35	0.91	0.74	1.11
High school graduate	**−0.53**	0.13	−3.94	0.00	0.59	0.45	0.77
College graduate	**−0.63**	0.17	−3.66	0.00	0.53	0.38	0.75
Married	−0.08	0.12	−0.66	0.51	0.92	0.73	1.17
Age < 50	−0.58	0.41	−1.42	0.16	0.56	0.25	1.25
Black	0.03	0.14	0.23	0.82	1.03	0.79	1.35
Hispanic	**0.53**	0.17	3.09	0.00	1.71	1.22	2.40
Other race	**0.46**	0.19	2.46	0.01	1.59	1.10	2.30
No insurance	0.17	0.31	0.55	0.58	1.19	0.65	2.17
Low income	**0.89**	0.14	6.39	0.00	2.44	1.86	3.21
Out of work	**0.76**	0.31	2.44	0.02	2.14	1.16	3.95
Out of the labor force	**0.91**	0.23	4.02	0.00	2.49	1.60	3.88
Age < 50 out of work	−0.48	0.57	−0.84	0.40	0.62	0.20	1.90
Age < 50 out of the labor force	0.57	0.44	1.29	0.20	1.77	0.74	4.22
Intercept	**−2.64**	0.31	−8.65	0.00	0.07	0.04	0.13
Difficulty doing errands alone such as visiting a doctor’s office or shopping							
		Std Err	t-Stat	*p* > |t|	Odds Ratio	95% Confidence Interval	
Female	**0.25**	0.09	2.89	0.00	1.29	1.08	1.53
High school graduate	**−0.48**	0.12	−3.92	0.00	0.62	0.49	0.79
College graduate	**−0.59**	0.15	−3.89	0.00	0.55	0.41	0.75
Married	**−0.19**	0.10	−1.95	0.05	0.82	0.68	1.00
Age < 50	−0.28	0.29	−0.95	0.34	0.76	0.43	1.34
Black	−0.03	0.13	−0.27	0.79	0.97	0.75	1.24
Hispanic	0.00	0.16	0.01	0.99	1.00	0.73	1.38
Other race	0.63	0.18	3.40	0.00	1.87	1.30	2.68
No insurance	0.19	0.20	0.94	0.35	1.21	0.81	1.81
Income < $50,000	**0.60**	0.13	4.74	0.00	1.82	1.42	2.33
Out of work	**0.73**	0.28	2.59	0.01	2.07	1.19	3.60
Out of the labor force	**1.27**	0.21	6.12	0.00	3.55	2.36	5.32
Age < 50 out of work	0.92	0.60	1.53	0.13	2.50	0.77	8.08
Age < 50 out of the labor force	**0.76**	0.34	2.27	0.02	2.15	1.11	4.15
Intercept	**−2.25**	0.28	−8.04	0.00	0.11	0.06	0.18

Bold Indicates significance at the 95% confidence level. Reference group: sex (male), race/ethnicity (White, non-Hispanic), age (≥50), marital status (not married), insurance status (not insured), income (≥$50,000), employment status (employed), education (less than high school).

**Table 3:** Poor physical/mental health among stroke survivors.

How many days did poor physical or mental health keep you from doing your usual activities, such as self-care, work, or recreation?							
		Std Err	t-Stat	*p* > |t|	IRR	95% Confidence Interval	
Female	−0.07	0.05	−1.36	0.17	0.94	0.85	1.03
High school graduate	**−0.25**	0.07	−3.79	0.00	0.78	0.68	0.89
College graduate	**−0.28**	0.09	−3.29	0.00	0.76	0.64	0.89
Married	−0.02	0.05	−0.34	0.74	0.98	0.88	1.09
Age < 50	−0.03	0.16	−0.18	0.86	0.97	0.70	1.34
Black	−0.12	0.08	−1.54	0.12	0.89	0.76	1.03
Hispanic	−0.09	0.10	−0.94	0.35	0.91	0.76	1.10
Other race	0.17	0.09	1.95	0.05	1.19	1.00	1.42
No insurance	0.17	0.11	1.55	0.12	1.18	0.96	1.46
Low income	**0.20**	0.07	2.78	0.01	1.23	1.06	1.41
Out of work	**0.73**	0.14	5.05	0.00	2.07	1.56	2.75
Out of the labor force	**0.54**	0.12	4.35	0.00	1.72	1.35	2.19
Age < 50 out of work	0.06	0.28	0.21	0.84	1.06	0.61	1.83
Age < 50 out of the labor force	**0.27**	0.18	2.54	0.01	1.32	1.23	1.86
Intercept	**1.96**	0.15	12.74	0.00	7.09	5.25	9.59
How many days during the past 30 days was your mental health not good?							
		Std Err	t-Stat	*p* > |t|	IRR	95% Confidence Interval	
Female	**0.22**	0.06	3.99	0.00	1.25	1.12	1.39
High school graduate	**−0.21**	0.08	−2.77	0.01	0.81	0.70	0.94
College graduate	**−0.46**	0.09	−5.01	0.00	0.63	0.53	0.75
Married	**−0.21**	0.06	−3.21	0.00	0.81	0.72	0.92
Age < 50	**0.31**	0.12	2.55	0.01	1.37	1.07	1.74
Black	−0.10	0.09	−1.12	0.26	0.91	0.76	1.08
Hispanic	−0.07	0.10	−0.74	0.46	0.93	0.77	1.13
Other race	0.09	0.09	0.96	0.34	1.09	0.91	1.32
No insurance	**0.20**	0.12	1.68	0.09	1.22	0.97	1.54
Low income	**0.22**	0.08	2.96	0.00	1.25	1.08	1.45
Out of work	0.25	0.15	1.67	0.09	1.29	0.96	1.72
Out of the labor force	−0.12	0.10	−1.14	0.26	0.89	0.73	1.09
Age < 50 out of work	0.14	0.26	0.55	0.58	1.15	0.70	1.90
Age < 50 out of the labor force	**0.24**	0.15	2.58	0.01	1.27	1.19	1.70
Intercept	**1.93**	0.15	13.20	0.00	6.91	5.19	9.20
How many days during the past 30 days was your physical health not good?							
		Std Err	t-Stat	*p* > |t|	IRR	95% Confidence Interval	
Female	0.03	0.04	0.84	0.40	1.04	0.95	1.12
High school graduate	**−0.14**	0.06	−2.45	0.01	0.87	0.78	0.97
College graduate	**−0.21**	0.07	−2.99	0.00	0.81	0.71	0.93
Married	−0.09	0.05	−1.87	0.06	0.92	0.84	1.00
Age < 50	−0.08	0.12	−0.71	0.48	0.92	0.73	1.16
Black	**−0.24**	0.06	−3.89	0.00	0.79	0.70	0.89
Hispanic	0.02	0.08	0.23	0.82	1.02	0.88	1.18
Other race	**0.18**	0.08	2.12	0.03	1.20	1.01	1.41
No insurance	**0.25**	0.11	2.36	0.02	1.28	1.04	1.58
Low income	**0.23**	0.06	3.79	0.00	1.26	1.12	1.41
Out of work	**0.51**	0.11	4.54	0.00	1.67	1.34	2.08
Out of the labor force	**0.38**	0.08	4.64	0.00	1.46	1.24	1.71
Age < 50 out of work	−0.09	0.29	−0.30	0.76	0.92	0.52	1.62
Age < 50 out of the labor force		0.14	2.09	0.28	1.26	1.19	1.53
Intercept	2.00	0.11	17.95	0.00	7.41	5.95	9.22

Bold Indicates significance at the 95% confidence level. Reference group: sex (male), race/ethnicity (White, non-Hispanic), age (≥50), marital status (not married), insurance status (not insured), income (≥$50,000), employment status (employed), education (less than high school).

## Data Availability

The original data presented in the study are openly available in BRFSS data at https://www.cdc.gov/brfss/index.html (accessed on 13 January 2026).
